# Attenuation of Autism-like Behaviors by an Anthocyanin-Rich Extract from Portuguese Blueberries via Microbiota–Gut–Brain Axis Modulation in a Valproic Acid Mouse Model

**DOI:** 10.3390/ijms23169259

**Published:** 2022-08-17

**Authors:** Diana Serra, Joana F. Henriques, Fábio J. Sousa, Mariana Laranjo, Rosa Resende, Marisa Ferreira-Marques, Victor de Freitas, Gabriela Silva, João Peça, Teresa C. P. Dinis, Leonor M. Almeida

**Affiliations:** 1 CNC—Center for Neuroscience and Cell Biology, University of Coimbra, 3004-531 Coimbra, Portugal; 2 Faculty of Pharmacy, University of Coimbra, 3004-531 Coimbra, Portugal; 3 CIVG—Vasco da Gama Research Center, EUVG—Vasco da Gama University School, 3020-210 Coimbra, Portugal; 4 PhD Program in Experimental Biology and Biomedicine (PDBEB), Institute for Interdisciplinary Research, University of Coimbra, 3004-531 Coimbra, Portugal; 5 REQUIMTE/LAQV—Research Unit, Faculty of Science, Porto University, 4099-002 Porto, Portugal; 6 Department of Life Science, Faculty of Science and Technology, University of Coimbra, 3004-531 Coimbra, Portugal

**Keywords:** Autism Spectrum Disorder, autism-like behaviors, anthocyanins, valproic acid, microbiota–gut–brain axis, gut microbiota, neuroinflammation

## Abstract

Autism Spectrum Disorders (ASDs) are a group of neurodevelopmental pathologies whose current treatment is neither curative nor effective. Anthocyanins are naturally occurring compounds abundant in blueberries and in other red fruits which have been shown to be successful in the treatment of several neurological diseases, at least in in vitro and in vivo disease models. The aim of the present work was to study the ability of an anthocyanin-rich extract (ARE) obtained from Portuguese blueberries to alleviate autism-like symptoms in a valproic acid (VPA) mouse model of ASD and to get insights into the underlying molecular mechanisms of such benefits. Therefore, pregnant BALB/c females were treated subcutaneously with a single dose of VPA (500 mg/kg) or saline on gestational day 12.5. Male offspring mice were orally treated with the ARE from Portuguese blueberries (30 mg/kg/day) or the vehicle for three weeks, and further subjected to behavioral tests and biochemical analysis. Our data suggested that the ARE treatment alleviated autism-like behaviors in in utero VPA-exposed mice and, at the same time, decreased both neuroinflammation and gut inflammation, modulated the gut microbiota composition, increased serotonin levels in cerebral prefrontal cortex and gut, and reduced the synaptic dysfunction verified in autistic mice. Overall, our work suggests that anthocyanins extracted from Portuguese blueberries could constitute an effective strategy to ameliorate typical autistic behaviors through modulation of the microbiota–gut–brain axis.

## 1. Introduction

Autism Spectrum Disorders (ASDs) are a group of neurodevelopmental conditions characterized by a deficit in communication and social interaction and by repetitive behaviors and restrictive interests [1]. Although ASDs are highly heritable disorders, several studies point to a complex genetic basis for its etiology [2] as well as to environmental factors, namely advanced parental age, maternal infection during pregnancy, and fetal exposure to insecticides or drugs, which together contribute to increase the risk [1]. On the other hand, similarly to other neurological disorders, such as Parkinson’s disease, the interplay between the gut or gut microbiota and the brain has recently gained much attention in the context of ASD on the basis of several pieces of evidence, namely: (i) children with ASD often report gastrointestinal symptoms (GI), such as diarrhea, constipation, and abdominal pain, and GI symptoms seem to be strongly correlated with the severity of children’s autistic behavior [3,4]; (ii) an exaggerated gut permeability occurs in 37% of ASD patients [5]; (iii) an increased gut and blood–brain-barrier permeability has been found in post-mortem samples from the brain and the gut of ASD patients [6]; (iv) ASD patients present neuroinflammation [7]; (v) ASD patients present gut dysbiosis [8]; (vi) prebiotic intervention and microbiota transfer therapy modulate gut microbiota and reduce autistic behavioral symptoms [9,10]. Since ASDs are lifelong disorders, whose current pharmacological treatment is only useful in managing co-morbid conditions, such as anxiety, bipolar disorder, and obsessive-compulsive behaviors [1], leading to severe side effects, innovative pharmacological strategies are required for the treatment of these disorders.

Polyphenols are a class of natural compounds easily obtained via dietary intake that have demonstrated to be highly efficient as anti-inflammatory and antioxidant agents in the scope of several inflammatory diseases [11]. Recently, studies carried out by Pereira et al. [12] and Serra et al. [13] showed that anthocyanins, a group of flavonoids abundant in red fruits, namely blueberries, can be envisaged as a promising strategy to reduce colitis in mice and microglia-driven neuroinflammation, respectively.

The present study aimed to evaluate the ability of an anthocyanin-rich extract to reduce autism-like behaviors via modulation of the communication between the gut and the brain, using the valproic acid (VPA)-treated mice model of ASD. VPA is an anti-epileptic drug whose administration during pregnancy, both in humans and in rodents, contributes to the development of autism in newborns, predominantly in males [14]. This model for ASD is particularly advantageous for the study of gut–brain communication in this context, since it can both mimic autistic-like behaviors and reproduce some of the GI alterations that have been described in autistic patients [14,15,16]. Since the mechanisms underlying VPA effect on the development of ASD are poorly understood, they will also be explored during this study.

## 2. Results

### 2.1. The Anthocyanin-Rich Extract Obtained from Portuguese Blueberries Has a High Content and a Great Diversity of Anthocyanins

The extract employed in this study showed a high content of anthocyanins, about 7.86 mg/mL, in terms of malvidin-3-glucoside, i.e., 131 mg per 100 g of blueberries. The total phenolic content of this fraction was 10.44 mg/mL, in terms of gallic acid equivalents, i.e., 174 mg per 100 g of blueberries.

**Figure 1:**
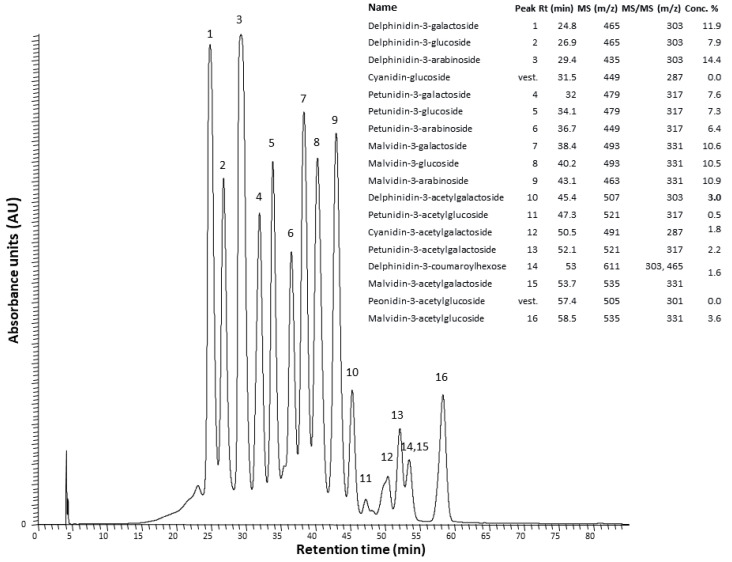
HPLC-DAD-MS/MS analysis of the anthocyanin-rich extract obtained from Portuguese blueberries. The peak identification and the contents of the different anthocyanins are indicated in the inset table. RT: retention time.

Moreover, a wide variety of anthocyanin molecules (16 peaks) was detected in ARE by HPLC-MS, as shown in Figure 1. The anthocyanins detected were, in descending order of amount, delphinidin, malvidin, petunidin, and cyanidin, conjugated with either arabinose, galactose, or glucose. Delphinidin-3-arabinoside (14.4%) and delphinidin-3-galactoside (11.9%) were the main anthocyanin components of ARE (Figure 1 inset).

### 2.2. The Anthocyanin-Rich Blueberry Extract Reduces Social Interaction Deficits and Repetitive Behaviors in in utero VPA-Exposed Offspring Mice

Prenatal VPA administration is associated with neurodevelopmental delay and an increased risk of ASD [14], with VPA-treated male mice showing decreased sociability and repetitive/stereotypic-like behaviors [17,18,19].

To determine the potential beneficial effects of ARE treatment in the altered behaviors reported in prenatal VPA-exposure male mice, we assessed motor function and social and stereotypical behaviors in adolescent mice.

During the behavior paradigms (P45–P55), in utero VPA-treated mice showed a slight tendency to weight loss compared to control mice. Additionally, no difference in the body weight was observed between the other experimental groups (Figure S1A—Supplementary data).

As abnormal motor activity has been reported in some ASD models, we tested whether VPA-exposed subjects exhibit altered locomotor activity in the open field test. We observed that the locomotor activity was not affected in VPA-exposed mice and that all groups travelled similar distances (Figure S1B—Supplementary data).

We used the three-chamber test to assess general sociability and interest in social novelty, as abnormal social behaviors have been largely reported in ASD mice models. In general, rodents tend to spend time with other rodents (Stranger 1) and investigate the novel stranger (Stranger 2) rather than the familiar one (Stranger 1) [20].

**Figure 2:**
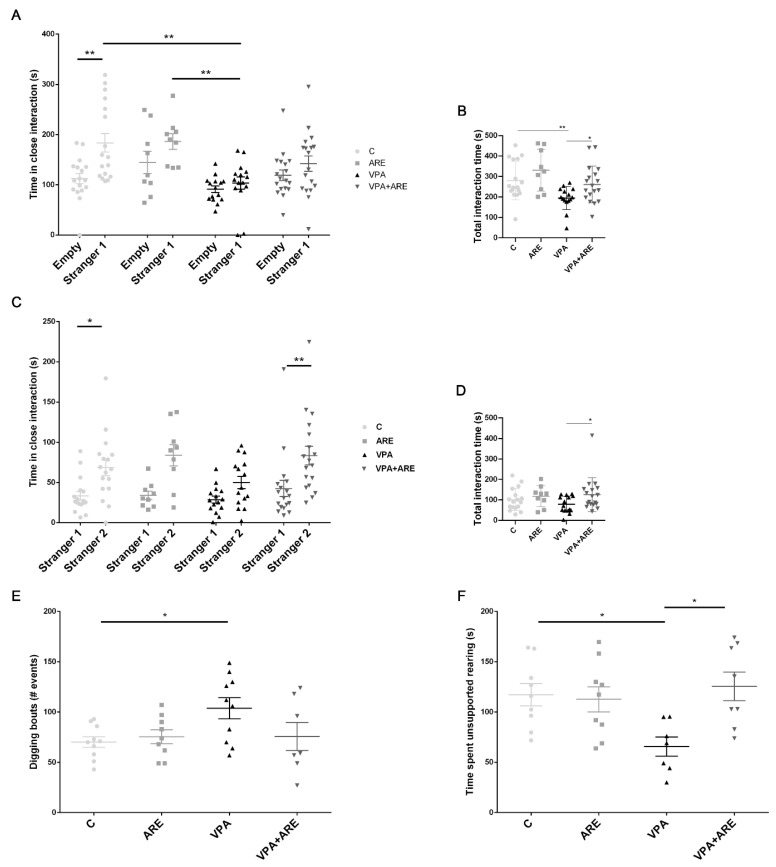
ARE treatment partially rescues social interactions in in utero VPA-treated mice. (**A**) In utero valproic acid (VPA)-treated mice showed sociability deficits by spending less time in social interaction (Stranger 1) than the control group (C) in the three-chamber social test trials. ARE treatment in mice prenatally exposed to VPA showed a tendency to rescue the sociability deficits observed in the VPA-treated group. (**B**) In utero VPA-treated mice spent reduced total interaction time in the sociability phase of the three-chambered test. ARE treatment led to a recovery of the total time spent in the three-chamber social test trial. (**C**) VPA-treated mice display a reduced tendency to engage in social interactions with the novel stranger (Stranger 2) compared to the control group. ARE administration led to an increase in social interaction with the unfamiliar mice (Stranger 2). (**D**) VPA-treated mice spend reduced total interaction time in the sociability phase of the novelty part. ARE treatment led to some recovery of this total interaction time. (**E**,**F**) VPA-treated group showed stereotypical behaviors, mainly increased number of digging bouts and decreased time in vertical explorations (rearing) in the homecage recordings. ARE treatment rescued the decreased rearing exploration (**F**) and did not significantly impact the digging (**E**). All data are presented as means ± SEM from 9–18 animals per group. Statistical significance: * *p* < 0.05 and ** *p* < 0.01.

In the sociability part of the test, the VPA-treated group spent less time in voluntary social interaction than the control group (Figure 2A), as quantified by total time in close interaction with the stranger animal. The 3-week daily oral administration of ARE in VPA-exposed male mice showed a tendency to restore the sociability deficits presented by the VPA-treated group. In contrast, ARE administration did not affect the control group (Figure 2A).

Accordingly, the social preference index revealed that ARE treatment induced no negative effect in the control group and slightly increased VPA-treated mice’s sociability (Figure S1C,D—Supplementary data). Additionally, the sum of total time spent in social interaction also showed that VPA-treated mice spent less time interacting in the sociability phase of the test (Figure 2B). Interestingly, VPA-exposed male mice with ARE treatment showed increased total interaction when compared to VPA-treated mice (Figure 2B).

In the social novelty part, when a novel social partner was introduced, the VPA-treated group tended to spend less time with the novel partner (Stranger 2) (Figure 2C) when compared to the control group (C—circles). Interestingly, VPA-exposed male mice with ARE treatment showed increased social interaction towards unfamiliar mice (Figure 2C). Here, the preference index shows that although VPA-treated mice preferred the unknown social partner, this preference is lower than in the control group, being the preference rescued by ARE treatment (Figure S1C,D—Supplementary data). Regarding the sum of total time spent in social interaction, VPA-exposed male mice with ARE treatment showed increased total time of interaction with social partners as compared to VPA-treated mice (Figure 2D).

Additionally, stereotypical behaviors, including grooming, digging, and rearing, were determined since these are considered core features of ASD-like dysfunction in mice [21]. The VPA-treated group showed an increased number of digging bouts and decreased time in vertical explorations (rearing) in the homecage recordings (Figure 2E,F).

Interestingly, the decrease in rearing exploration was rescued by ARE treatment (Figure 2F), where no significant difference in digging was observed in the same experimental group (Figure 2E). No difference was observed in grooming levels in these animals (Figure S1E—Supplementary data).

Overall, these observations suggest that in utero exposure to VPA leads to deficits that resemble autism, such as altered social behavior and repetitive behaviors in male mice. The oral administration of ARE rescues some of these altered behaviors, being the positive impacts more robust in the ability to engage in (novel) social interactions and to ameliorate repetitive behaviors, mainly excessive vertical plane explorations in the homecage context.

### 2.3. The Anthocyanin-Rich Blueberry Extract Treatment Is Efficient in Reducing Neuroinflammation in in utero VPA-Exposed Offspring Mice

To assess the potential of ARE in reducing neuroinflammation in in utero VPA-exposed offspring mice, the mRNA levels of microglia activation markers were monitored, microglia morphology alterations were evaluated, and the number of Iba-1^+^ microglial cells was counted in the cerebral cortex of mice.

**Figure 3:**
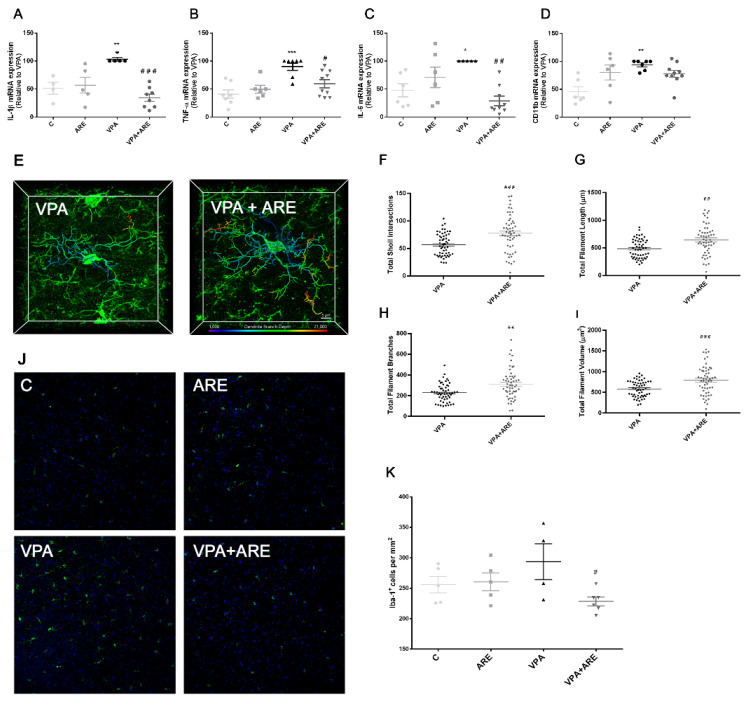
Neuroinflammation exhibited in mice prenatally exposed to VPA is reduced by the anthocyanin-rich extract. (**A**–**D**) mRNA levels of microglia activation markers were evaluated in the cerebral cortex of mice by RT-PCR, as described in “Materials and Methods”. ARE treatment led to a significant decrease of neuroinflammatory markers levels in mice prenatally exposed to VPA. All data are presented as means ± SEM from 4–8 animals per group. * *p* < 0.05, ** *p* < 0.01, and *** *p* < 0.001 vs. control mice and ^#^ *p* < 0.05, ^##^ *p* < 0.01, and ^###^ *p* < 0.001 vs. in utero VPA-exposed mice. (**E**) Representative confocal microscopy pictures of microglial 3D morphological images of the cerebral cortex of in utero VPA-exposed mice treated and untreated with ARE. (**F**–**I**) Total sholl intersections and total filament branches, length, and volume were analyzed after data collection from Imaris software, as described in “Materials and Methods”. ARE treated in utero VPA-exposed offspring mice led to a significant increase in total sholl intersections, total filament length, total filament branches, and total filament volume, as compared to mice not treated with ARE. Values are means ± SEM from 4 animals per group. ^##^ *p* < 0.01 and ^###^ *p* < 0.001 vs. in utero VPA-exposed mice. (**J**) Representative confocal microscopy pictures of Iba1 positive cells. Simultaneous DNA labeling with Hoechst was performed to visualize the nuclear compartments. (**K**)**.** The number of Iba1 positive cells was evaluated in the cerebral cortex of mice by immunofluorescence and confocal microscopy, as described in “Materials and Methods”. ARE treatment led to a reduction of the number of Iba1 positive cells present in the cerebral cortex of in utero VPA-exposed mice. Values are means ± SEM from 4–6 animals per group. ^#^ *p* < 0.05 vs. in utero VPA-exposed mice.

As pictured in Figure 3A–D, in utero VPA-exposed offspring mice demonstrated a significant mRNA production of microglia activation markers, namely IL-1β, TNF-α, IL-6, and CD11b, and, as expected, daily ARE treatment of in utero VPA-exposed offspring mice, for three weeks, significantly downregulated the expression of these microglia activation markers. To support the efficiency of ARE to counteract neuroinflammation, microglia morphology was monitored in mice treated and untreated with ARE through confocal microscopy (Figure 3E–I). Activated microglia, de-ramified and presenting large and short dendrites, were observed in in utero VPA-exposed mice (Figure 3E–I), in accordance with previous descriptions of microglia morphology in neuroinflammatory conditions [22]. However, ARE treated in utero VPA-exposed offspring mice showed microglial cells with a significant increase in total sholl intersections, total filament length, total filament branches, and total filament volume, as compared to mice not treated with ARE, thus indicating that ARE promotes the transition from an activated phenotype to a “resting” (but surveillant) state of microglial cells (Figure 3E–I).

Regarding Iba-1, a common marker of activated microglia, a significant decrease was found in the number of Iba-1^+^ microglial cells in the cerebral cortex of in utero VPA-exposed mice treated with ARE, as compared to mice not treated with ARE, as illustrated in Figure 3J–K.

### 2.4. The Anthocyanin-Rich Blueberry Extract Treatment Decreases Gut Inflammation and Modulates Gut Microbiota Composition in in utero VPA-Exposed Offspring Mice

To investigate whether ARE was able to ameliorate gut inflammation and to modify gut microbiota composition in in utero VPA-exposed mice, mRNA levels of important pro-inflammatory markers were quantified in the mice’s ileum, and the gut microbiota composition was fully characterized by DNA sequencing.

**Figure 4:**
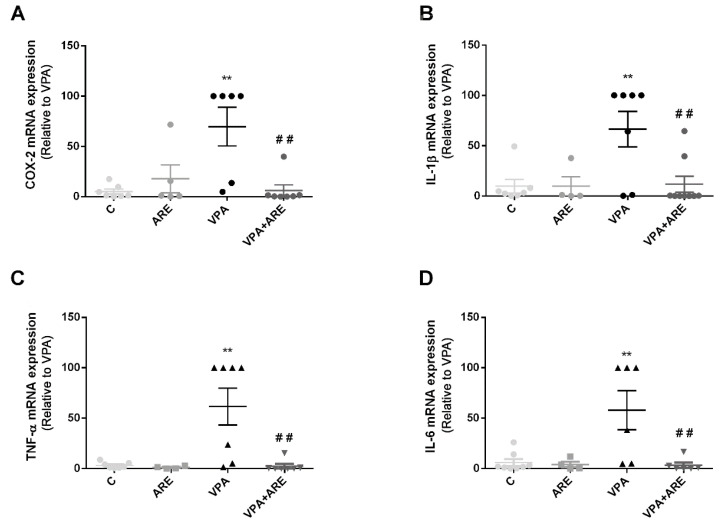
Gut inflammation observed in mice prenatally exposed to VPA is attenuated by the anthocyanin-rich extract. (**A**–**D**) mRNA levels of pro-inflammatory cytokines were evaluated in the ileum of mice by RT-PCR, as described in “Materials and Methods”. ARE treatment significantly reduced the levels of inflammatory mediators present in the gut of in utero VPA-exposed mice. Values are means ± SEM from 4–9 animals per group. ** *p* < 0.01 vs. control mice and ^##^ *p* < 0.01 vs. in utero VPA-exposed mice.

As depicted in Figure 4A–D, mRNA levels of COX-2, IL-1β, TNF-α, and IL-6 in in utero VPA-exposed mice were significantly higher than those of control mice (without in utero exposure to VPA). Upon daily treatment with ARE, for three weeks, a significant reduction in the mRNA levels of those critical pro-inflammatory mediators was observed in prenatally VPA-exposed mice, as compared with the untreated mice.

**Figure 5:**
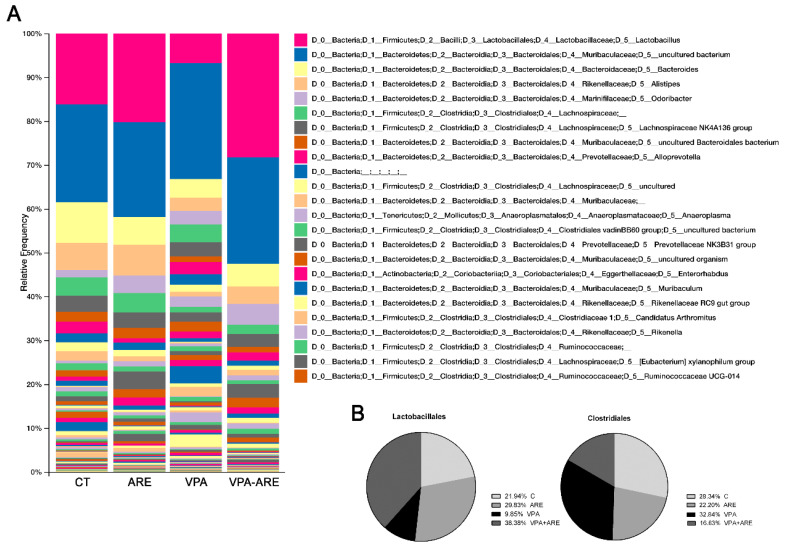
Gut microbiota dysregulation observed in mice prenatally exposed to VPA is modulated by the anthocyanin-rich extract. (**A**) Bar graph summarizing the relative abundance of microbial composition of mice. (**B**) Circular graphs depicting the percentage of Lactobacillales and Clostridiales in the gut of mice prenatally exposed to VPA or not and treated or non-treated with ARE. ARE treatment led to an increase of Lactobacillales abundance and to a reduction of Clostridiales abundance when compared to VPA-exposed mice non-treated with ARE.

Additionally, as illustrated in Figure 5A,B, it was observed that in utero VPA-exposed mice showed a high abundance of Clostridiales, in contrast, there was a low abundance of Lactobacillales and ARE treatment was able to modulate gut microbiota composition, especially increasing Lactobacillales abundance and decreasing Clostridiales population.

### 2.5. The Anthocyanin-Rich Blueberry Extract Treatment Promotes the Production of Serotonin in the Prefrontal Cortex, in the Ileum, and in the Colon of in utero VPA-Exposed Offspring Mice

**Figure 6:**
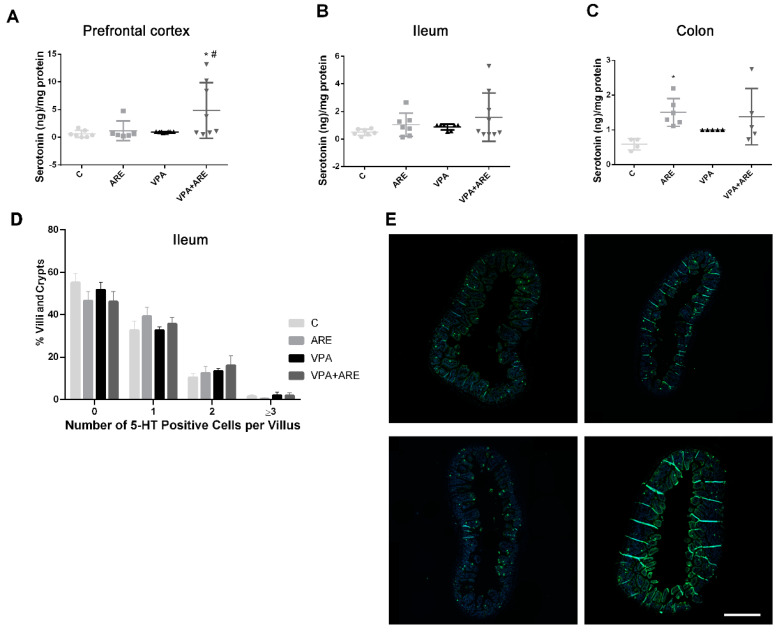
Serotonin levels is significantly increased in in utero VPA-exposed mice treated with the anthocyanin-rich extract. (**A**–**C**) Serotonin levels were evaluated in the prefrontal cortex, in the ileum, and in the colon of mice by ELISA, as described in “Materials and Methods”. ARE treatment led to an increase of serotonin levels in the prefrontal cortex and in the gut (ileum and colon) of in utero VPA-exposed mice. Values are means ± SEM from at least 4–9 animals per group. * *p* < 0.05 vs. control mice and ^#^ *p* < 0.05 vs. in utero VPA-exposed mice. (**D**). The number of serotonin (5-HT) positive cells per villi and crypts were evaluated in the ileum of mice by immunofluorescence and confocal microscopy, as described in “Materials and Methods”. ARE treatment led to an increment of the number of 5-HT positive cells in the villi and crypts of the ileum of prenatally VPA-exposed mice. Values are means ± SEM from 7–10 animals per group (**E**). Representative confocal microscopy pictures of 5-HT positive cells.

Since serotonin is a neurotransmitter that is 95% produced in the gut and some bacteria present in the gut microbiota can modulate serotonin levels, the levels of this neurotransmitter were evaluated in the brain and in the gut of mice treated and untreated with ARE. As depicted in Figure 6A–C, ARE treatment increased the serotonin levels both in the prefrontal cortex and in the gut (ileum and colon) of in utero VPA-exposed mice, although more significantly in the prefrontal cortex of mice. The results observed in Figure 6D,E corroborate the results patent in Figure 6B, since the number of cells producing serotonin found in the ileum, per villi and crypt, was higher in in utero VPA-exposed mice treated with ARE as compared to untreated mice.

### 2.6. The Anthocyanin-Rich Blueberry Extract Treatment Decreases Synaptic Pruning Dysregulation in in utero VPA-exposed Offspring Mice

**Figure 7:**
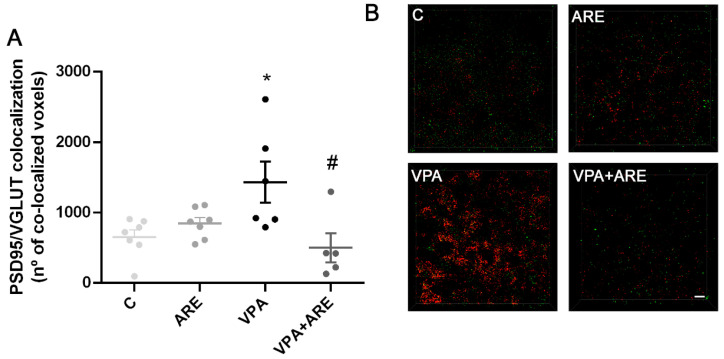
Neuronal excitability is significantly reduced in in utero VPA-exposed mice treated with the anthocyanin-rich extract. (**A**) PSD95/VGLUT colocalization was evaluated by immunofluorescence, as described in “Materials and Methods”. ARE treatment led to a significant reduction of the neuronal excitability verified in in utero VPA-exposed mice. Values are means ± SEM from 5–7 animals per group. * *p* < 0.05 vs. control mice and ^#^ *p* < 0.05 vs. in utero VPA-exposed mice. (**B**). Representative confocal microscopy pictures of PSD95/VGLUT colocalization.

Since microglia cells play an essential role in eliminating excessive synapses for normal brain development, and synaptic pruning dysfunction has been associated with impairment of social behavior [23], it seemed pertinent to assess the effect of prenatal VPA exposure on synaptic pruning. As illustrated in Figure 7A,B, in utero VPA-exposed mice showed a significant increase in the colocalization of PSD95/VGLUT (key proteins involved in excitatory synaptic transmission), suggesting that VPA promoted an impairment in synaptic pruning in in utero VPA-exposed offspring mice.

In contrast, ARE treatment was demonstrated to be effective in reducing the synaptic dysfunction verified in in utero VPA-exposed mice (Figure 7A,B), as denoted by its ability to reduce the excessive synapses observed in in utero VPA-exposed mice.

## 3. Discussion

In the present study, an anthocyanin-rich extract (ARE) obtained from Portuguese blueberries was used, for the first time, as a therapeutic approach in a mouse model of ASD. Unlike other polyphenols, such as resveratrol and curcumin, anthocyanins have never been tested in ASD, and for this reason, this work is pioneering. Furthermore, this work was focused on clarifying the molecular mechanisms underlying the ARE effects in ASD context, particularly concerning the microbiota-gut-brain axis, which constitutes a novel strategy in this field.

The study began with the preparation of ARE from Portuguese blueberries and its chemical characterization. HPLC analysis showed that delphinidin 3-arabinoside was the most abundant anthocyanin in this ARE, although many other anthocyanins were found, such as malvidin, petunidin, and cyanidin derivatives. Then, this chemically characterized ARE was administered to in utero VPA-exposed male mice to evaluate its ability to reduce autism-like behaviors, such as altered social behavior and repetitive behaviors. The oral administration of ARE rescued some of these altered behaviors, the positive impacts being more robust in the ability to engage in (novel) social interactions and to ameliorate repetitive behaviors, mainly excessive vertical plane explorations in the homecage context. Since social interaction impairments and repetitive behaviors are both common patterns of ASD [1], compounds capable of reducing these typical autistic behaviors, such as anthocyanins, hold great promise in ASD treatment.

Further, the following steps of this work were focused on evaluating the molecular mechanisms underlying ARE effects on the amelioration of autistic-like behaviors in mice, with particularly interest in microbiota–gut–brain communication. Microbiota–gut–brain dysfunction in ASD has not been clarified yet, but it is now accepted that neuroinflammation is probably linked to gut inflammation/gut microbiota dysregulation, which are common features of many neurological diseases, including ASD [24]. Therefore, the ability of ARE to reduce both neuro- and gut inflammation and to modulate the gut microbiota composition was evaluated. ARE treatment was shown to be effective in lowering VPA-mediated neuroinflammation in in utero VPA-exposed mice, namely via downregulating key pro-inflammatory cytokines, such as TNF-α, IL-1β, and IL-6, decreasing the number of Iba-1^+^ cells, i.e., activated microglia cells, and increasing the surveillant phenotype of microglial cells in the cerebral cortex of mice. These results are in complete accordance with data obtained by Bhandari et al. [25], revealing that a different polyphenolic compound, resveratrol, reduced neuroinflammation in a propionic acid rat model of ASD.

Regarding gut inflammation, it is noteworthy that VPA administered prenatally in mice provoked a significant increase in pro-inflammatory cytokines, such as COX-2, IL-1β, TNF-α, and IL-6, in the ileum of offspring mice as well as dysbiosis, characterized by an enhancement in Clostridiales population and a reduction of Lactobacillales population. This increase of Clostridiales observed in mice prenatally exposed to VPA is consistent with data reported by Theije et al. [19]. Accordingly, Luna et al. [26] also reported an increase of Clostridiales in ASD children with functional gastrointestinal disorders, suggesting that it was correlated to the production of inflammatory cytokines as well as to clinical symptoms in ASD patients. The reduction of Lactobacillales abundance, reported in this study in prenatally VPA exposed mice, is also in agreement with data obtained by Kong et al. [27] showing a similar decay in Lactobacillaceae in autistic mice as compared to control groups.

ARE treatment of in utero VPA-exposed mice was shown to be able not only to decrease gut inflammation but also to regulate gut microbiota composition, increasing the abundance of Lactobacillales and decreasing that of Clostridiales population. It is well known that the beneficial effect of Lactobacillales is related to its ability to reinforce gut epithelial integrity [28] and to induce the production of anti-inflammatory cytokines [29]. Accordingly, some in vivo studies claim that *Lactobacillus plantarum* and *Lactobacillus brevis* improve intestinal inflammation by downregulation of TLR4/NF-kB signaling pathway [30,31]. In contrast, Clostridiales abundance contributes to a detrimental effect on intestinal inflammation [29]. For example, *Clostridium difficile* is a type of bacteria that can promote a serious inflammation of the colon and whose infection worsens Inflammatory Bowel Disease outcome. The pathogenicity of *Clostridium difficile* is probably related to its capacity to increase the levels of pro-inflammatory cytokines, such as TNF-α and IFN-γ [32].

Therefore, the increment in gut Lactobaccillales and the reduction in Clostridiales, resulting from the ARE treatment in autistic mice, may have contributed to the attenuation of gut inflammation verified in ARE-treated mice. Furthermore, since the severity of gastrointestinal symptoms in children with ASD has been correlated to the severity of their autistic behaviors [3,33], it is licit to infer that the effect of ARE treatment on reducing gut inflammation could have promoted the improvement of autistic-like behaviors of in utero VPA-stimulated mice, as mentioned before. This agrees with a recent double-blind, randomized, and placebo-controlled trial performed in Taiwan, which suggested that *Lactobaccillus plantarum* PS128 ameliorated some autistic symptoms in young ASD children [34].

Although, in this study, we only focused on the beneficial effect of ARE extract on the modulation of bacterial species from gut microbiota, it is known that despite being less abundant, fungi are important components of human gut microbiota and that fungal dysbiosis may have impact on human health [35]. Therefore, it would be interesting to explore the potential effect of ARE extract on fungal composition of autistic mice in a near future.

On the other hand, the hypothesis of serotonin involvement in the development of ASD has also been proposed, although controversial data have been collected [36]. In the present work, VPA-stimulated “autistic” mice did not present noticeable differences in terms of serotonin production as compared to control mice, neither in prefrontal cortex, nor in ileum or colon tissues. Nevertheless, ARE treatment showed a somewhat increase of serotonin levels both in the gut and in the brain of in utero VPA-exposed mice.

It is noteworthy that the majority of serotonin of the human body is produced in the gut, mainly by the enterochromaffin cells (EC). These cells are endocrine cells especially sensitive to mechanical and chemical stimulus, whose identification is still under study [37]. It has been reported that gut microorganisms, such as *Lactobaccillus plantarum* and *Lactobaccillus lactis*, can increase serotonin levels by inducing the secretion of serotonin by EC cells or incrementing the production of serotonin from tryptophan, the main precursor of serotonin synthesis [38]. Therefore, in this study, the increase in serotonin biosynthesis verified in the gut of prenatally VPA-exposed mice can be related, either to the ability of ARE to increase Lactobaccillales abundance, stimulating then EC cells, as mentioned before, or to the increase of tryptophan availability for serotonin production. In fact, recently, our research group reported the presence of tryptophan in significant amounts in a similar ARE extract by HPLC-MS analysis [13]. Regarding the augmentation of serotonin production in the prefrontal cortex of VPA-exposed mice promoted by ARE treatment, this can be explained by the permeation either of serotonin, coming from the gut, or of tryptophan, through the blood–brain-barrier (BBB), to be converted to serotonin. This hypothesis is not plausible in a normal situation unless the BBB is partially leaky, as happened in neuroinflammation [39]. Very recently, Walsh et al. [40] suggested that the enhancement of serotonin signaling reverses social deficits in several mouse models of ASD, and for this reason, it seems reasonable that the enhancement of serotonin levels in in utero VPA-exposed mice treated with ARE has contributed to the amelioration of autism-like behaviors observed in these mice.

Finally, to identify further mechanisms underscoring ARE’s valuable effect on attenuation of autistic-like behavior, it seemed pertinent to investigate whether synaptic pruning was affected by in utero VPA exposure of mice and by ARE treatment. Synaptic pruning is an essential process managed by microglial cells for normal brain development [41] and its impairments have been described in some neurodevelopmental disorders, including ASD [42]. In this work, it was observed that synaptic pruning was impaired in mice prenatally exposed to VPA and ARE treatment efficiently reduced the excessive synapses observed in those mice, reducing the colocalization of PSD95 and VGLUT in the cortex of mice. The reduction of synaptic pruning dysfunction may have also contributed to attenuating autistic-like behaviors in ARE-treated mice. This is coherent with Zhan et al. [23] reporting that deficits in synaptic pruning are associated with deficits in social interaction and with increased repetitive behaviors in mice.

In conclusion, the present study contributed to the clarification of the mechanisms underlying VPA effect on the development of ASD and, at the same time, it demonstrated that ARE treatment can ameliorate autism-like behaviors in mice prenatally exposed to VPA, through modulation of the microbiota–gut–brain communication. This can be inferred through ARE ability to (i) reduce neuroinflammation; (ii) decrease gut inflammation and regulate gut microbiota composition; (iii) increase serotonin levels in the brain and in the gut; and (iv) reverse synaptic pruning dysfunction in mice prenatally exposed to VPA.

Therefore, this ARE obtained from Portuguese blueberries was shown to be a promising therapeutic strategy to alleviate autistic symptoms in mice and opens the window for the implementation of innovative therapies to treat ASD patients.

## 4. Materials and Methods

### 4.1. Animal Model and Experimental Design

**Figure 8:**
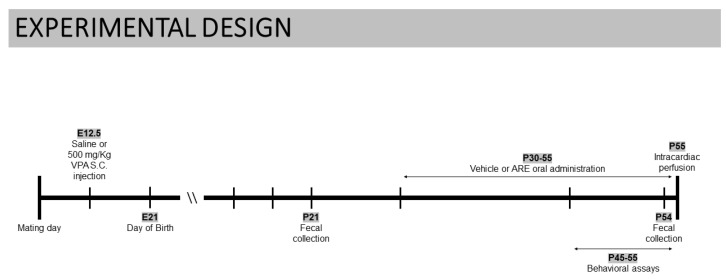
Schematic of experimental design.

BALB/c breeding pairs from Charles River Laboratories and their offspring were maintained at a constant temperature (22 °C) and humidity (60%), under a 12 h light/dark cycle in an individual cage ventilation system. Animals had free access to water and food ad libitum. Maintenance and handling of the animals was performed in compliance with all relevant ethical animal testing and research regulations, including the Animals Use and Care Guidelines issued by FELASA and European Directives on Animal Welfare. All experiments with mice were carried out under animal testing research protocols approved by ORBEA (Animal Welfare Committee of the Centre for Neuroscience and Cell Biology and of the Faculty of Medicine, University of Coimbra, reference number 253/2020) and by DGAV (Portuguese Regulatory Agency, reference number 003897/2020). The experimental design, whose main stages are illustrated in Figure 8, was as follows: females were mated for 36 h and pregnant females were treated subcutaneously with 500 mg/kg VPA, purchased by Sigma Chemicals Co. (St. Louis, MO, USA) or sterile saline on embryonic day 12.5 (E12.5). The dose of VPA was selected according to previous studies showing that this dose was less harmful for the pregnant females and that it was appropriate to induce the same behavioral abnormalities [43]. Offspring were housed with their mother until weaning on postnatal day 21 (P21).

To determine the effects of oral administration of an anthocyanin-rich blueberry extract (ARE), in utero VPA- or saline-exposed male offspring were subdivided and daily orally exposed to a subtoxic concentration of ARE (30 mg/kg), incorporated in commercially available gelatin or to an ARE-free gelatin for 3 weeks (P30–55). Male offspring were subjected to several behavioral tests (P45–55) performed by trained experimentalists blinded to animal treatments. Fecal pellets from animals placed individually in a clean cage were collected at the beginning and at the end of the ARE treatment (P21 and P54, respectively). Body weight was monitored twice/week throughout the study.

### 4.2. Plant Material Collection and Preparation of the Anthocyanin-Rich Extract

Blueberries (*Vaccinium corymbosum* L., Bluecrop cultivar), from organic farming, were collected at the time of peak production in the central region of Portugal (Sever do Vouga, Aveiro, Portugal) and kept at −80 °C until use.

The anthocyanin-rich extract (ARE) was prepared as previously described [13] and kept at −80 °C under nitrogen until analysis and use [12]. The protocol was adapted according to Oszmianski et al. [44] and modified in line with Youdim et al. [45].

### 4.3. Chemical Characterization of the Anthocyanin-Rich Extract

The quantification of total phenolic content was performed using the Folin–Ciocalteau reagent from Sigma Chemicals Co., as described by Georgé et al. [46]. Results were expressed as milligrams of gallic acid equivalents (GAE) per volume of ARE (mL) and as milligrams of GAE per 100 g of blueberries. Total monomeric anthocyanins in the ARE were estimated spectrophotometrically using a pH differential method described by Giusti et al. [47]. The results were expressed as grams of malv3glc equivalents per volume (mL) of ARE and as milligrams of malv3glc equivalents per 100 g blueberries.

HPLC analysis of the anthocyanin was performed after separation on a liquid chromatograph (Hewlett-Packard 1100 series, HP, Palo Alto, CA, USA) equipped with an AQUATM (Phenomenex, Torrance, CA, USA) reversed-phase column (150 × 4.6 mm, 5 mm, C18), thermostated at 35 °C. The solvents were A: H_2_O:HCOOH _(9:1)_ and B: H_2_O:CH_3_CN:HCOOH _(6:3:1)_ in a gradient of 20–52.5% B for 35 min at a flow rate of 1.0 mL min^−1^. The column was washed with 100% B for 15 min and then stabilized at the initial conditions for another 15 min.

Double online detection was done in a photodiode spectrophotometer and by mass spectrometry. The mass detector was a Finnigan LCQ (Finnigan Corporation, San Jose, CA, USA) equipped with an atmospheric pressure ionization (API) source, using an electrospray ionization (ESI) interface. Both the auxiliary and the sheath gas were a mixture of nitrogen and helium. The capillary voltage was 3 V and the capillary temperature 190 °C. Spectra were recorded in positive ion mode between m/z 120 and 1500. The mass spectrometer was programmed to do a series of three scans: a full mass, a zoom scan of the most intense ion in the first scan, and a MS-MS of the most intense ion using relative collision energies of 30 and 60.

### 4.4. Behavioral Tests

#### 4.4.1. Three-Chamber Social Interaction Test

The three-chamber arena was from Stoelting (Dublin, Ireland). Male offspring were tested for voluntary social interaction as previously described [48,49]. The assay consisted of three sessions: the first session began with a 20-min habituation period during which the subject mouse freely explored all three chambers. Next, the mouse was confined to the center chamber, and an empty wire cage (Empty—‘E’) and a cage with an unfamiliar mouse (Stranger 1—‘S1’) were introduced to the side-chambers. In the second session, the subject mouse was allowed to freely explore all three chambers for 20 min. Before the third and last session, the subject mouse was gently guided to the center chamber while the empty wire cage was replaced with a caged unfamiliar stimulus mouse (Stranger 2—‘S2’). In the last session, the subject mouse was left to explore all three chambers for 10 min. Stimulus mice were males of the same age, weight-matched, previously habituated to the wire cages. The positions of the empty cage and ‘S1’ were alternated between tests. No position bias was observed. Time spent in proximity and distance travelled were calculated using the automated software EthoVision XT v.11.0 software (Noldus, Wageningen, The Netherlands). Preference index for each mouse was calculated as the time spent in close proximity with the familiar partners (S1) divided by the total time spent in close proximity to the familiar partner and empty chamber (S1 + E) or as the time spent in close proximity with the novel partners (S2) divided by the total time spent in close proximity to the familiar and novel partners (S1 + S2).

#### 4.4.2. Repetitive Behaviors Quantification

Animals were placed in a separate standard cage with bedding and recorded for 30 min. Quantified behaviors included digging, grooming, and unsupported rearing were manually scored by an observer blinded to the treatment of the mice. Digging was defined as mice using their paws to pull dirt or cage bedding backwards. Unsupported rearing was scored as fast, rhythmic movement of the forepaws while the mouse stands in an upright position. Quantification was performed off-line using The Observer XT 12 software (Noldus, Wageningen, The Netherlands)

#### 4.4.3. Open Field Test

In the open field (40 × 40 × 30 cm) test, each animal was placed at the corner of the apparatus and locomotor activity as recorded for 1 h by video-tracked using EthoVision XT v.11.0 software (Noldus, Wageningen, The Netherlands). Homogeneous and indirect illumination of the room was provided by white LED lamps at 100 lx. Distance travelled was evaluated by automatic quantification.

### 4.5. Quantitative Real-Time RT-PCR (qRT-PCR) for Gene Expression

At the end of the experiment, animals were transcardially perfused. Cerebral cortex and ileum samples were collected, cryoprotected, and stored at −80 °C until processed. Total RNA was extracted from cerebral cortex and ileum tissue samples by using the RNA extraction kit Aurum^TM^ Total RNA Mini (Bio-Rad, Hercules, CA, USA, 7326820,), according to the manufacturer’s instructions. Complementary DNA synthesis was performed using 1 µg of total RNA [deoxyribonuclease I (DNase I)–treated] with NZY First-Strand cDNA Synthesis Kit (NZYTech, Lisboa, Portugal, MB12501). The primers for TNF-α, IL-1β, IL-6, CD11b, and COX-2, as well as the housekeeping genes HPRT-1 (hypoxanthine phosphoribosyltransferase-1) and actin were designed using the Beacon Designer 8.2 software (PREMIER Biosoft International, Palo Alto, CA, USA) and purchased from NZYTech (Lisboa, Portugal). The primers sequences are represented in the Table 1, presented below:

The efficiency of the amplification reaction for each gene was calculated by running a standard curve of serially diluted cDNA sample. Gene expression was analyzed using iQ or iTaq SYBR Green Supermix on the CFX Connect Real-Time PCR Detection System (Bio-Rad, Hercules, CA, USA). The results for each gene of interest were normalized against the housekeeping genes HPRT-1 or actin, found to be stable under our experimental conditions, and expressed as a percentage of positive control using the Livak method.

### 4.6. Detection of Serotonin Levels

To measure serotonin levels, prefrontal cortex, ileum, and colon tissues were washed in sterile saline and homogenized with RIPA lysis buffer in a particular ratio (15 mg of tissue for 150 µL of lysis buffer). Samples were kept at −20 °C until processing according to the Elisa kit manufacturer’s protocol (Enzo Life Sciences, Farmingdale, NY, USA ADI-900-175). The competitive serotonin ELISA Assay kit uses the microtiter plate format. Standards and samples are added to a GxR IgG-coated plate and a solution of serotonin conjugated to alkaline phosphatase is then added to the plate, followed by a solution of rabbit polyclonal antibody, specific to serotonin. The antibody binds, in a competitive manner, to the serotonin in the sample or in the conjugate. Being a competitive assay, the obtained signal is inversely proportional to the amount of serotonin in the sample. The sensitivity of the kit is 0.293 ng/mL. Protein concentrations were calculated using a BCA assay (Thermo Fisher Scientific, Waltham, MA, USA).

### 4.7. Immunofluorescence for Iba-1^+^ Cells, 5-HT^+^ Cells and PSD95/VGLUT Colocalization

Brain and intestinal tissues were collected after animal perfusion with ice-cold phosphate-buffered saline (PBS). Brain hemispheres were fixed by immersion in 4% paraformaldehyde in PBS overnight. After that, brains were washed with PBS and then cryoprotected using a sucrose gradient (15 and 30%). After 24 h, brains were mounted in optimal cutting temperature (OCT) embedding medium, frozen, and cryosectioned in the cryostat (Cryostar NX50, Thermo Fisher Scientific, Waltham, MA, USA). Coronal sections from brains (20 µm thickness) were collected on SuperFrost^©^ microscope slides (Thermo Fisher Scientific, Waltham, MA, USA) and stored at −20 °C. Paraffin-embedding sections of ileum were submitted to deparaffinization and rehydration procedures. Frozen sections were defrosted by at least 60 min and hydrated with PBS for 10 min. After hydration, slides were treated for antigen retrieval following three cycles of microwave treatment (400 W; 4 min) with 0.01 M citrate buffer (pH 6.0). Sections were permeabilized with 0.25% Triton X-100 for 15 min, washed with PBS for 10 min, and blocked with 5% bovine serum albumin (BSA), and 5% fetal bovine serum (FBS) in PBS for 60 min in a humid chamber at room temperature. Brain sections were further incubated with rabbit-derived anti-Iba-1 (1:1000, Fujifilm Wako, Bellwood Road, Richmond, VA, USA, 019-19741) or rabbit-derived anti-PSD-95 (1:200, Cell Signaling Technology, Danvers, MA, USA, 1673409S) and guinea pig-derived anti-VGLUT (1:500, Synaptic Systems, Göttingen, Germany, 135304) in blocking solution in a humidified chamber overnight (two overnights for anti-Iba-1) at 4 °C. For ileum sections, rabbit-derived anti-serotonin (1:5000, Sigma Chemicals Co., St. Louis, MO, USA, S5545) was diluted in a blocking solution and incubated in a humidified chamber overnight at 4 °C. Then, sections were incubated for 60 min with secondary antibody donkey anti-rabbit Alexa Fluor 488 (1:500 or 1:1000, Thermo Fisher Scientific, Waltham, MA, USA, A-11008) or goat anti-guinea pig Alexa Fluor 594 (1:500, Thermo Fisher Scientific, Waltham, MA, USA, A-11076). After the secondary antibody, sections were profoundly washed and incubated for 10 min with Hoechst 33258 (1:1000, Sigma Chemicals Co., St. Louis, MO, USA, 94403). Slides were coverslipped using Fluoroshield^TM^ (Sigma Chemicals Co., St. Louis, MO, USA, F6182) and visualized under a confocal microscope Zeiss LSM 710 confocal microscope.

### 4.8. Analysis for Microglial Morphology

For microglial 3D morphological reconstruction, Z-stack images (30 µm depth, 1 µm step at 40× magnification) of the cerebral cortex were obtained using a confocal microscope Zeiss LSM710 with a Plan-Apochromat 40×/1.4 Oil DIC M27 objective (1024 × 1024 pixel, 16-bit depth, pixel size 0.63 µm, zoom 0.7). A total of six images per animal across three different sections of the cerebral cortex were randomly acquired for microglial morphology analysis. Raw.czi files were converted for IMARIS 9.6 software (Oxford Instruments). For all analyses, 5–6 40× z-stacks were used to reconstruct 12–18 cells per animal. All images were acquired using the same settings and all images were reconstructed by a researcher blinded to the experimental conditions.

### 4.9. Faecal Sample Preparation and Sequencing/Microbiome Profiling

DNA was extracted from 40 mouse feces samples using QIAamp Powerfecal Pro DNA Kit (Qiagen, Hilden, Germany), according to the manufacturer’s instructions with minor modifications. All samples were quantified, and four equimolar pools were prepared: control mice, without exposure to VPA (C); control mice not treated with VPA but treated with ARE (ARE); in utero VPA-exposed mice (VPA); and in utero VPA-exposed mice submitted to the daily treatment with ARE, for 3 weeks (VPA plus ARE).

Samples were prepared for Illumina Sequencing by 16S rRNA gene amplification of the bacterial community. The DNA was amplified for the hypervariable V3-V4 region with specific primers and further reamplified in a limited-cycle PCR reaction to add sequencing adapters and dual indexes. Initial PCR reactions were performed for each sample using KAPA HiFi HotStart PCR Kit (Qiagen) according to manufacturer instructions, with a 0.3 μM concentration for each PCR primer: forward primer Bakt_341F 5′–CCTACGGGNGGCWGCAG-3′ and reverse primer Bakt_805R 5′–GACTACHVGGGTATCTAATCC-3′ [50,51] and 2.5 μL of pooled DNA in a total volume of 25 μL. The PCR conditions involved a 3 min denaturation at 95 °C, followed by 25 cycles of 98 °C for 20 s, 55 °C for 30 s and 72 °C for 30 s and a final extension at 72 °C for 5 min. Second PCR reactions added indexes and sequencing adapters to both ends of the amplified target region according to manufacturer’s recommendations (Illumina, 2013). Negative PCR controls were included for all amplification procedures. PCR products were then one-step purified and normalized using SequalPrep 96-well plate kit (ThermoFisher Scientific, Waltham, MA, USA) [52], pooled and pair-end sequenced in the Illumina MiSeq^®^ sequencer with the Miseq Reagent kit v3 (600 cycles), according to manufacturer’s instructions (Illumina, San Diego, CA, USA) at Genoinseq (Cantanhede, Portugal).

Sequence data were processed at Genoinseq (Cantanhede, Portugal). Raw reads were extracted from Illumina MiSeq^®^ System in a fastq format and quality-filtered with PRINSEQ version 0.20.4 [53] to remove sequencing adapters, trim bases with an average quality lower than Q25 in a window of 5 bases, and eliminate reads with fewer than 150 bases. The forward and reverse reads were merged by overlapping paired-end reads with AdapterRemoval version 2.1.5 [54] using default parameters. The QIIME2 package version 2020.2 [55,56] is a powerful tool used for Operational Taxonomic Unit (OTU) generation, taxonomic identification, sample diversity, and richness indices calculation. This bioinformatic software helps researchers in the comprehensive analysis of microbiome sequence data to generate publishable figures from raw data. Sample IDs were assigned to the merged reads and converted to fasta format. Chimeric merged reads were detected and removed using VSearch [57], an implementation of UCHIME [58] against the SILVA database version 132 [59]. OTUs were selected at 97% similarity threshold using the open reference strategy and those with less than two reads were removed from the OTU table. A representative sequence of each OTU was then selected for taxonomy assignment using the referred database.

### 4.10. Statistical Analysis

All data were expressed as means ± SEM of at least 4 animals per group. Differences between groups were analyzed by one-way or two-way analysis of variance (ANOVA), Tukey’s and two-tailed Student *t*-test were used as appropriate. All statistical analysis was carried out with the GraphPad Prism 8 software (San Diego, CA, USA) and values of *p* < 0.05 were accepted as statistically significant.

## Figures and Tables

**Table 1:** Primers used for qRT-PCR (F: Forward; R: Reverse).

**TNF-α**	F: 5′-AGTCTGTATCCTTCTAAC-3′	R: 5′-TTCTGAGTAGTTGTTGAA-3′
**IL-1β**	F: 5′-CAATGGACAGAATATCAAC-3′	R: 5′-ACAGGACAGGTATAGATT-3′
**IL-6**	F: 5′-ACCAAGACCATCCAATTCAT-3′	R: 5′-GCTTAGGCATAACGCACTA-3′
**CD11b**	F: 5′-ACCAGTGATGAGAGTTCTAT-3′	R: 5′-CTGATGCTGTATGACCTTAC-3′
**COX-2**	F: 5′-ATCAGACCTTCCTTGTAT-3′	R: 5′-CACACTCATAGTTAAGACA-3′
**HPRT-1**	F: 5′-CCATTCCTATGACTGTAGA-3′	R: 5′-CTTCAACAATCAAGACATTC-3′
**Actin**	F: 5′-ATCTTCCGCCTTAATACT-3′	R: 5′-GCCTTCATACATCAAGTT-3′

## Data Availability

Not applicable.

## Supplementary Materials

The following supporting information can be downloaded at: https://www.mdpi.com/article/10.3390/ijms23169259/s1.
