# Supplementary material for: Attenuation of Autism-like Behaviors by an Anthocyanin-Rich Extract from Portuguese Blueberries via Microbiota–Gut–Brain Axis Modulation in a Valproic Acid Mouse Model
Source: Int J Mol Sci. 2022 Aug 17;23(16):9259. doi: 10.3390/ijms23169259 (PMC9409076; doi:10.3390/ijms23169259)
Supplement: Supplementary file 1 [file ijms-23-09259-s001.zip › ijms-1857658-supplementary.pdf]

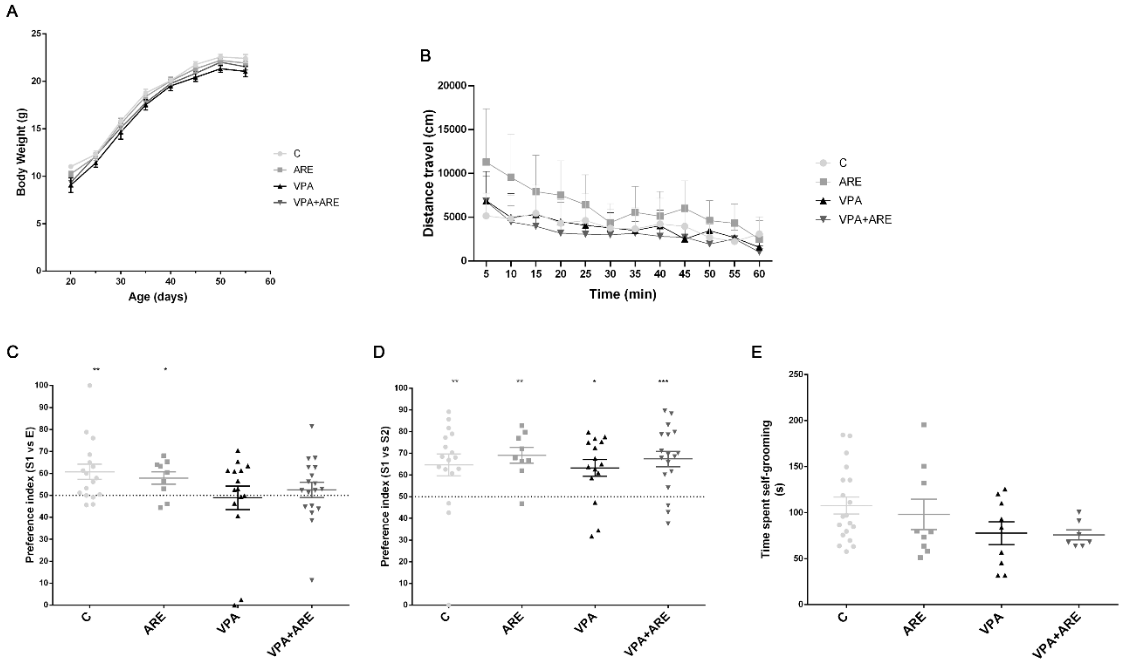

Figure S1. Characterization of in utero VPA-exposed mice (VPA) and in utero VPA-exposed mice treated with ARE. **(A).** VPA-treated mice showed a slight tendency to decrease body weight compared to VPA-treated mice. No body weight difference was observed between the other experimental group. **(B).** No altered locomotor activity in VPA-exposed mice in the open field test. The total distance travelled by the four experimental groups is similar. **(C).** VPA-exposed mice show no preference index for a social partner in the sociability phase of the test. VPA-exposed mice treated with ARE have a slightly increased number of social interactions**. (D)**. VPA-exposed mice show a reduced preference for social interaction with a novel stranger. ARE administration increased the preference index for novel social interactions. (**E).** No difference in grooming time. All data are presented as means ± SEM from 5-18 animals per group. Statistical significance: $*p < 0.05$, $**p < 0.01$ and $***p < 0.001$.
